# miR-181c-5p Exacerbates Hypoxia/Reoxygenation-Induced Cardiomyocyte Apoptosis via Targeting PTPN4

**DOI:** 10.1155/2019/1957920

**Published:** 2019-04-17

**Authors:** Liang Ge, Yin Cai, Fan Ying, Hao Liu, Dengwen Zhang, Yanjing He, Lei Pang, Dan Yan, Aimin Xu, Haichun Ma, Zhengyuan Xia

**Affiliations:** ^1^Department of Anesthesiology, The First Hospital, Jilin University, Jilin, China; ^2^State Key Laboratory of Pharmaceutical Biotechnology, The University of Hong Kong, Hong Kong; ^3^Department of Anesthesiology, The University of Hong Kong, Hong Kong; ^4^Department of Cardiology, The Second Affiliated Hospital of Guangzhou Medical University, Guangzhou Institute of Cardiovascular Disease, Guangdong, China; ^5^Department of Anesthesiology, Guangdong Provincial People's Hospital, Guangdong Academy of Medical Sciences, Guangzhou, Guangdong, China

## Abstract

**Background:**

Activation of cell apoptosis is a major form of cell death during myocardial ischemia/reperfusion injury (I/RI). Therefore, examining ways to control cell apoptosis has important clinical significance for improving postischemic recovery. Clinical evidence demonstrated that miR-181c-5p was significantly upregulated in the early phase of myocardial infarction. However, whether or not miR-181c-5p mediates cardiac I/RI through cell apoptosis pathway is unknown. Thus, the present study is aimed at investigating the role and the possible mechanism of miR-181c-5p in apoptosis during I/R injury by using H9C2 cardiomyocytes.

**Methods and Results:**

The rat origin H9C2 cardiomyocytes were subjected to hypoxia/reoxygenation (H/R, 6 hours hypoxia followed by 6 hours reoxygenation) to induce cell injury. The results showed that H/R significantly increased the expression of miR-181c-5p but not miR-181c-3p in H9C2 cells. In line with this, in an *in vivo* rat cardiac I/RI model, miR-181c-5p expression was also significantly increased. The overexpression of miR-181c-5p by its agomir transfection significantly aggravated H/R-induced cell injury (increased lactate dehydrogenase level and reduced cell viability) and exacerbated H/R-induced cell apoptosis (greater cleaved caspases 3 expression, Bax/Bcl-2 and more TUNEL-positive cells). In contrast, inhibition of miR-181c-5p *in vitro* had the opposite effect. By using computational prediction algorithms, protein tyrosine phosphatase nonreceptor type 4 (PTPN4) was predicted as a potential target gene of miR-181c-5p and was verified by the luciferase reporter assay. The overexpression of miR-181c-5p significantly attenuated the mRNA and protein expression of PTPN4 in H9C2 cardiomyocytes. Moreover, knockdown of PTPN4 significantly aggravated H/R-induced enhancement of LDH level, cleaved caspase 3 expression, and apoptotic cell death, which mimicked the proapoptotic effects of miR-181c-5p in H9C2 cardiomyocytes.

**Conclusions:**

These findings suggested that miR-181c-5p exacerbates H/R-induced cardiomyocyte injury and apoptosis *via* targeting PTPN4 and that miR-181c-5p/PTPN4 signaling may yield novel strategies to combat myocardial I/R injury.

## 1. Introduction

Ischemic heart disease caused by the narrowness or occlusion of myocardial coronary arteries is one of the leading causes of morbidity and mortality worldwide. In the clinical setting, the most effective intervention to reduce ischemic injury is the timely restoration of coronary blood flow, but reperfusion itself may lead to additional tissue damage and pathological remodeling, which is termed “myocardial ischemia/reperfusion (I/R) injury” [1, 2]. Despite the fact that molecular mechanisms mediating myocardial I/R injury are largely unknown, activation of cell apoptosis has been suggested to play an important role in its pathology [3, 4]. Apoptosis, a process of programmed cell death, significantly contributes to both cardiomyocyte loss during myocardial I/R injury and postischemic adverse cardiac remodeling [5]. Accumulating evidence has also demonstrated that cardiomyocyte apoptosis occurs predominantly in the surviving portion of the ischemic myocardium [6]. Also, the morbidity of symptomatic heart failure after acute myocardial infarction in patients was positively associated with increased myocardial apoptosis [7]. Thus, inhibition of excessive apoptosis in cardiomyocytes can be an effective approach to combat ischemic heart disease.

MicroRNAs (miRNAs) are endogenous noncoding small RNA molecules with about 21-24 nucleotides and induce potent gene silencing by complementarily pairing with the 3′-untranslated region (3′-UTR) of the target mRNA, therefore playing important roles in various cellular and biological processes, such as ischemic cardiomyopathy [8], cardiac remodeling [9], heart failure [10], and arrhythmia [11]. The miR-181 family contains four miRNAs (miR-181a, miR-181b, miR-181c, and miR-181d), which were found to be highly homologous and widely distributed in the body [12]. Emerging evidence has suggested that miR-181 family members are also highly present in the heart [13]. Intriguingly, recent study demonstrated that different miR-181 family members have divergent effects on myocardial function [14]. In particular, miR-181a/b has been shown to mediate cardioprotection against myocardial I/R injury that was associated with the suppression of cytosolic phosphatase and tensin homolog expression, whereas miR-181c/d targeted mitochondrial cytochrome c oxidase 1 to reduce myocardial I/R injury as shown in miR-181c/d knockout mice [14]. In line with this, clinical evidence demonstrated that miR-181c was significantly upregulated in the myocardium in patients with congenital heart defects [15]. Furthermore, miR-181c level was also significantly upregulated in the failing heart or under hypoxia condition, which was positively correlated with the severity of apoptosis [13]. However, whether or not miR-181c exacerbates cardiac I/R injury through cell apoptosis and in particular the underlying mechanism is unknown. In addition, since mature miRNAs can be generated from the opposite arms of the same precursor of miRNA [16], the two mature miR-181c that synthesize from 5′ or 3′ arm of the precursor are denoted with miR-181c-5p (named miR-181c in other studies) and miR-181c-3p (a passenger miRNA of miR-181c), respectively. Given the underestimated physiological relevance of passenger miRNAs [17, 18], the present study is aimed at investigating which strand of the mature miR-181c (5p or 3p or both) was altered in the context of myocardial I/R injury and to determine the molecular basis of potential cause-effect link between miR-181c expression and apoptosis during ischemia using rat origin cardiomyocytes (H9C2) subjected to hypoxia/reoxygenation (H/R).

## 2. Materials and Methods

### 2.1. H9C2 Cell Culture and Treatment

The rat cardiomyocyte-derived cell line H9C2 obtained from the American Type Culture Collection (ATCC, VA, USA) was cultured in Dulbecco's modified Eagle's medium (DMEM, Thermo Fisher Scientific, MA, USA) supplemented with 10% fetal bovine serum (FBS, Biosera, MO, USA) and 1% penicillin/streptomycin (PS, 100 U/mL, Thermo Fisher Scientific). The H9C2 cardiomyocytes were incubated at 37°C in a room air atmosphere containing 5% CO_2_-95% O_2_. Rat origin H9C2 cardiomyocytes were seeded into six-well plate (2 × 10^5^ cell/well) overnight and randomly divided into the following four groups: (1) control (CTL)+negative control (NC) agomir or NC antagomir or scramble siRNA; (2) CTL+miR-181c-5p agomir or miR-181c-5p antagomir or PTPN4 siRNA; (3) H/R+NC agomir or NC antagomir or scramble siRNA; and (4) H/R+miR-181c-5p agomir or miR-181c-5p antagomir or PTPN4 siRNA. H/R stimulation was achieved as previously described [4]. Briefly, the H9C2 cardiomyocytes were incubated in DMEM medium (no glucose or serum) and placed on a humidified Plexiglas chamber containing 95% N_2_ and 5% CO_2_ to mimic ischemia. After six hours, the cells were exposed to normal medium (DMEM+FBS+PS) and room air atmosphere containing 5% CO_2_ and 95% O_2_ for additional six hours.

### 2.2. *In Vivo* Coronary Ligation Model and Infarct Size Determination

Male adult Sprague-Dawley rats (220 ± 20 g, 8 weeks of age) were obtained from and housed in the Laboratory Animal Unit (The University of Hong Kong). All rats were fed with standard chow on a 12-hour light/dark cycle with water provided *ad libitum*. The *in vivo* myocardial I/R model was induced by occluding the left anterior descending artery for 30 min followed by 2 hours of reperfusion as previously described [19]. Sham operations will be performed by passing a thread under the coronary artery without ligation. Myocardial infarct size (IS) was determined by using Evans blue/TTC (1% 2,3,5-triphenyltetrazolium chloride) staining as previously described [20]. IS was expressed as a percentage of the area at risk (AAR). All experimental procedures were approved by The University of Hong Kong Committee on the Use of Live Animals for Teaching and Research.

### 2.3. MicroRNA and Small Interfering RNA Transfection

Rno-miR-181c-5p agomir, rno-miR-181c-5p antagomir, and small interfering RNA (siRNA) against –PTPN4 and their negative controls were synthesized by RiboBio (Guangdong, China). H9C2 cells in exponential phase of growth were plated in six-well plates and cultured overnight. The cells were then transfected with the miR-181c-5p agomir or antagomir (50 nM), siRNA (50 nM), or their negative controls (50 nM) using Lipofectamine 2000 (Invitrogen, CA, USA) for 24 hours according to the manufacturer's protocol. The effects of transfection efficiency were validated by measuring the expression of miRNA-181c-5p or the mRNA expression of PTPN4 through real-time polymerase chain reaction (PCR).

### 2.4. Analysis of LDH Leakage and Cell Viability

Cell injury was assessed by the biomarker lactate dehydrogenase (LDH). The released LDH in the collected medium was measured by using a commercial LDH kit (Roche, Germany) according to the manufacturer's instructions. The cell viability of the H9C2 cells in different groups was determined by the 3-(4,5-dimethylthiazol-2-yl)-2,5-diphenyltetrazolium bromide (MTT) assay as previously described [4]. Briefly, the H9C2 cells seeded in 96-well plates with different treatments were incubated with MTT solution (1 mg/mL, Sigma-Aldrich, MO, USA) at 37°C for four hours. The formazan crystals were dissolved with dimethyl sulfoxide (DMSO, 100 mL/well), and the absorbance was detected at 570 nm with Epoch microplate spectrophotometer (BioTek, VT, USA).

### 2.5. Luciferase Reporter Assay

A pmiR-RB-REPORT™ vector was constructed by inserting a fragment of the 3′-UTR of rat PTPN4 mRNA containing the putative miR-181c-5p binding site (position 4722-6401) which was purchased from RiboBio. As a mutated vector, the mutations in the seed binding sites of 3′-UTR fragment of rat PTPN4 (4915-4921 and 6333-6339) were generated from TGAATGT to ACTTACT. For reporter assay, 293T cells were transfected with 100 ng of wild-type or mutated plasmid and 50 nM of miR-181c-5p agomir or negative control using Lipofectamine 3000 (Invitrogen). Firefly and Renilla luciferase activities were measured 24 hours posttransfection using the Dual-Glo™ Luciferase Assay System (Promega).

### 2.6. Real-Time Polymerase Chain Reaction

Total RNA was extracted from H9C2 cardiomyocytes or heart tissues using RNAiso Plus (Takara, Japan) according to the manufacturer's instructions. Equal amounts of total RNA were reverse-transcribed to first-strand cDNA using the PrimeScript RT Master Mix Kit (Takara), according to the manufacturer's instructions. For reverse transcription of miRNAs, specific Bulge-Loop™ miRNA primers (RiboBio) were used instead of the Oligo dT primers in the PrimeScript™ RT reagent kit (Takara). Quantitative real-time PCR was performed as previously described [21] using SYBR Green master mix (Takara) on an Applied Biosystems Prism 7000 sequence detection system (Applied Biosystems). The conditions for amplification were 30 seconds at 95°C for denaturation, 40 cycles of 5 seconds at 95°C, and 30 seconds at 60°C. The primer sequences used are listed in Table 1. Relative mRNA or miRNA levels were analyzed by using the 2^-ΔΔCt^ method and normalized to those of *β*-actin or 5S, respectively.

### 2.7. Western Blotting

Total cell lysates were collected by lysing H9C2 cardiomyocytes with lysis buffer supplemented with Protease Inhibitor Cocktail (Thermo Fisher Scientific) and Phosphatase Inhibitor Cocktail (Roche). The protein concentration of the cell lysate was measured by Bradford assay (Bio-Rad, CA, USA). The extracted protein samples were separated by 8%–12.5% sodium dodecyl sulfate polyacrylamide gel electrophoresis and transferred onto polyvinylidene difluoride membranes for detection with appropriate antibodies. Primary antibodies against total caspase 3 (1 : 1000), cleaved caspase 3 (1 : 1000), Bax (1 : 1000), Bcl-2 (1 : 1000), GADPH antibody (1 : 1000), and horseradish peroxidase-conjugated anti-mouse or anti-rabbit secondary antibodies (1 : 3000) were purchased from Cell Signaling Technology. Anti-Ptpn4 antibody (1 : 1000) was purchased from Novus. Blots were visualized with Clarity ECL Western Blotting Detection Reagent (Bio-Rad) and subsequently exposed to X-ray film (Carestream, NY, USA). ImageJ software (National Institutes of Health, MD, USA) was used to analyze the optical densities of the immunoreactive bands. Protein expression was normalized to that of loading control (GAPDH).

### 2.8. TUNEL Assay

H9C2 cardiomyocytes were cultured directly on the chamber slides (Thermo Fisher Scientific) at 1 × 10^4^ cells/chamber. After the various treatments, the apoptotic cells were determined by TUNEL (terminal deoxynucleotidyl transferase dUTP nick end labeling) staining using an In Situ Cell Death Detection Kit (Roche), according to the manufacturer's instructions. Images were captured with an Olympus BX41 fluorescence microscope equipped with an OlympusDP72 color digital camera (Olympus). Five photos (magnification ×200) were taken randomly for each sample. Image quantification was presented as percentage of TUNEL-positive cells among the total number of cells as previously described [4].

### 2.9. Statistical Analysis

All data are expressed as the means ± SEM. Comparison between groups was carried out by two-tailed unpaired Student's *t*-test, Mann–Whitney test, or two-way ANOVA followed by Bonferroni's test, wherever appropriate, using the GraphPad Prism 7.0 software (San Diego, CA, USA). *P* values less than 0.05 were considered statistically significant differences.

## 3. Results

### 3.1. miR-181c-5p Is Upregulated in H/R-Stimulated H9C2 Cardiomyocytes and in the Postischemic Myocardium of Rats

**Figure 1:**
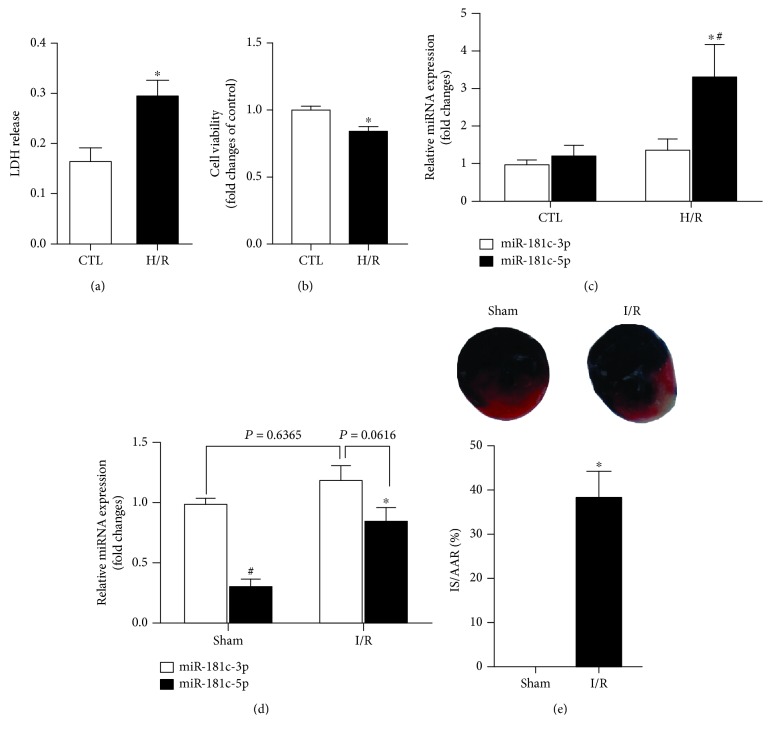
Hypoxia/reoxygenation (H/R) enhanced LDH release (a) and reduced cell viability (b) that was concomitant with significantly enhanced expression of miR-181c-5p but not miR-181c-3p (c) in H9C2 cardiomyocytes. In in vivo studies, myocardial I/R (induced by 30 minutes of left anterior descending artery occlusion and 2 hours of reperfusion in rats) induced significant increase in expression of miR-181c-5p but not in miR-181c-3p (d) and increased postischemic myocardial infarction (e). Data are shown as means ± SEM; ^∗^*P* < 0.05 vs. CTL or sham, *n* = 5.

To mimic the ischemia/reperfusion condition *in vivo*, the H9C2 cardiomyocytes were subjected to six-hour hyperoxia, followed by six-hour reoxygenation. In response to H/R stimulation, there was significantly enhanced level of LDH release and reduced cell viability when compared with the control group, suggesting the establishment of H/R-stimulated injury model in cardiomyocytes (Figures 1(a) and 1(b)). There was no significant difference between the expression of miR-181c-3p and miR-181c-5p in unstimulated H9C2 cardiomyocytes. However, the expression of post-H/R cardiomyocyte miR-181c-5p was significantly increased by 118% as compared to unstimulated H9C2 cardiomyocytes (Figure 1(c)), whereas the expression of miR-181c-3p did not significantly change between control and H/R groups (Figure 1(c)). Of note, the cardiac level of miR-181c-5p was significantly reduced as compared to the expression of miR-181c-3p in the rats with sham operation (Figure 1(d)), which was different from that seen in the H9C2 cardiomyocytes. In the *in vivo* myocardial I/R model (30 min left anterior descending coronary artery ligation followed by two hours of reperfusion), the increase of postischemic myocardial infarction size (Figure 1(e)) was accompanied by significantly increased expression of miR-181c-5p but not miR-181c-3p as compared to those in the sham group (Figure 1(d)). Taken together, these findings suggested that the upregulation of miR-181c-5p may be involved in the pathology of myocardial I/R injury.

### 3.2. Overexpression of miR-181c-5p Aggravated H/R-Induced Cell Injury and Apoptosis in H9C2 Cardiomyocytes

**Figure 2:**
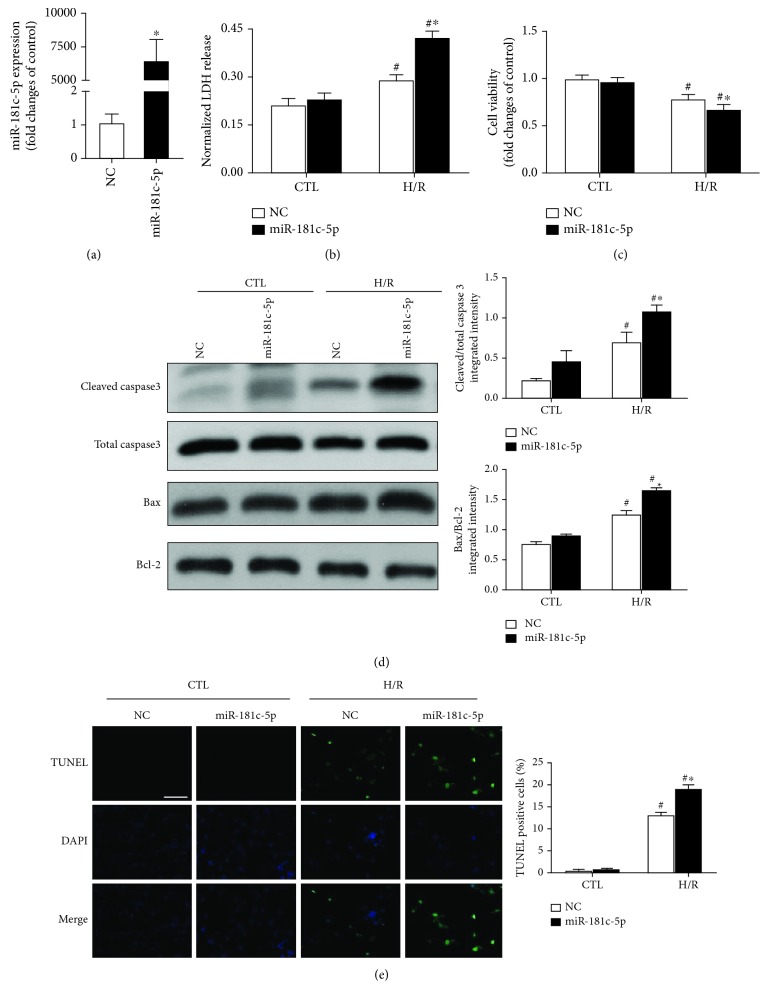
Transfection of cells with miR-181c-5p agomir (miR-181c-5p) resulted in significant overexpression of miR-181c-5p in H9C2 cardiomyocytes (a), and overexpression of miR-181c-5p exacerbated the H/R-induced H9C2 cell injury, as evidenced by further increased LDH release (b), reduced cell viability (c), and increased apoptotic cell death as evidenced by increased cleaved caspase 3 and Bax/Bcl-2 (d) and increased TUNEL-positive cells (e). Scale bar: 200 *μ*m. Data are shown as means ± SEM; ^#^*P* < 0.05 vs. CTL, ^∗^*P* < 0.05 vs. NC agomir (NC), *n* = 6.

To investigate the role of miR-181c-5p in H/R-induced cell injury and apoptosis, gain-of-function experiments were performed in H9C2 cardiomyocytes. The overexpression of miR-181c-5p model was established by transfection of miR-181c-5p agomir into H9C2 cardiomyocytes, which resulted in an approximately 6000-fold increase of miR-181c-5p level when compared to mock-transfected (NC agomir) cells (Figure 2(a)). H/R-stimulated induction of LDH leakage, which is a biomarker of cell injury, was further significantly increased in H9C2 cardiomyocytes in the presence of miR-181c-5p overexpression (Figure 2(b)). Consistently, the cell viability after H/R stimulation was significantly reduced in the presence of miR-181c-5p overexpression in H9C2 cardiomyocytes (Figure 2(c)).

Apoptosis is a major form of cardiomyocyte death during I/R injury [22]. Thus, it is tempting to speculate that overexpression of miR-181c-5p may attenuate H/R-induced cell apoptosis. To verify this hypothesis, the apoptosis-related proteins, including Bax, Bcl-2, and total and cleaved caspase 3, were detected. As anticipated, the ratios of Bax/Bcl2 and cleaved/total caspase 3 were significantly enhanced in the H/R-stimulated cells when compared with the control group. In response to H/R stimulation, the overexpression of miR-181c-5p resulted in the greater ratios of Bax/Bcl-2 and cleaved/total caspase3 (Figure 2(d)), leading to significant augmentation of H/R-stimulated increment of apoptotic cell death as measured by TUNEL assay (Figure 2(e)), which detects extensive DNA degradation during the late stages of apoptosis [23]. Taken in conjunction, these observations suggest that overexpression of miR-181c-5p aggravates H/R-induced cell injury and apoptosis in H9C2 cardiomyocytes.

### 3.3. Inhibition of miR-181c-5p Alleviated H/R-Induced Cell Injury and Apoptosis in H9C2 Cardiomyocytes

**Figure 3:**
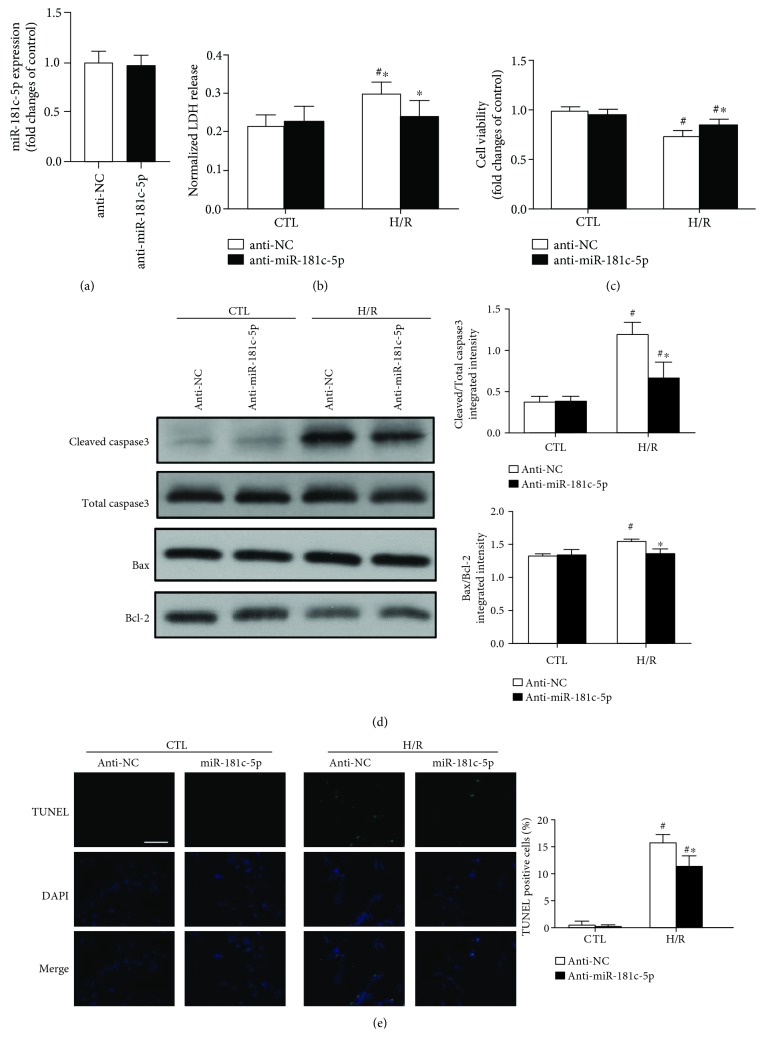
Inhibition of miR-181c-5p with miR-181c-5p antagomir (anti-miR-181c-5p) did not change the expression of miR-181c-5p (a) but alleviated H/R-induced H9C2 cell injury (as evidenced by reduced LDH release (b) and increased cell viability (c)) and reduced apoptosis (as evidenced by reduced ratios of cleaved/total caspase 3 and Bax/Bcl-2 (d) and attenuated TUNEL-positive apoptotic cells (e)). Scale bar: 200 *μ*m. Data are shown as means ± SEM; ^#^*P* < 0.05 vs. CTL, ^∗^*P* < 0.05 vs. NC antagomir (anti-NC), *n* = 6.

To consolidate the role of miR-181c-5p in H/R-induced cell injury and apoptosis, experiments were performed in H9C2 cardiomyocytes transfected with miR-181c-5p antagomir. An antagomir is a small synthetic RNA, which is chemically modified and perfectly complementary to the specific miRNA target. Through binding to the miR-181c-5p, miR-181c-5p antagomir can prevent the direct interaction between miRNA and its target mRNA and thus inhibit the ability of miR-181c-5p but not induce its degradation. As anticipated, there was no significant difference in the level of miR-181c-5p between anti-NC and anti-miR-181c-5p groups (Figure 3(a)). Inhibition of miR-181c-5p significantly alleviated H/R-induced cell injury, as evidenced by reduced LDH leakage (by 24%, Figure 3(b)) and increased cell viability (by 16%, Figure 3(c)), when compared to H/R-stimulated H9C2 cardiomyocytes transfected with negative control of antagomir. In contrast to the result in the miR-181c-5p overexpression cardiomyocytes, inhibition of miR-181c-5p significantly attenuated the ratios of Bax/Bcl-2 and cleaved/total caspase3 (Figure 3(d)) and the numbers of TUNEL-positive apoptotic cells (Figure 3(e)) in response to H/R stimulation. Taken together, these results indicate that inhibition of miR-181c-5p alleviates H/R-induced cell injury and apoptosis in H9C2 cardiomyocytes.

### 3.4. miR-181c-5p Targeted Protein Tyrosine Phosphatase Nonreceptor Type 4

**Figure 4:**
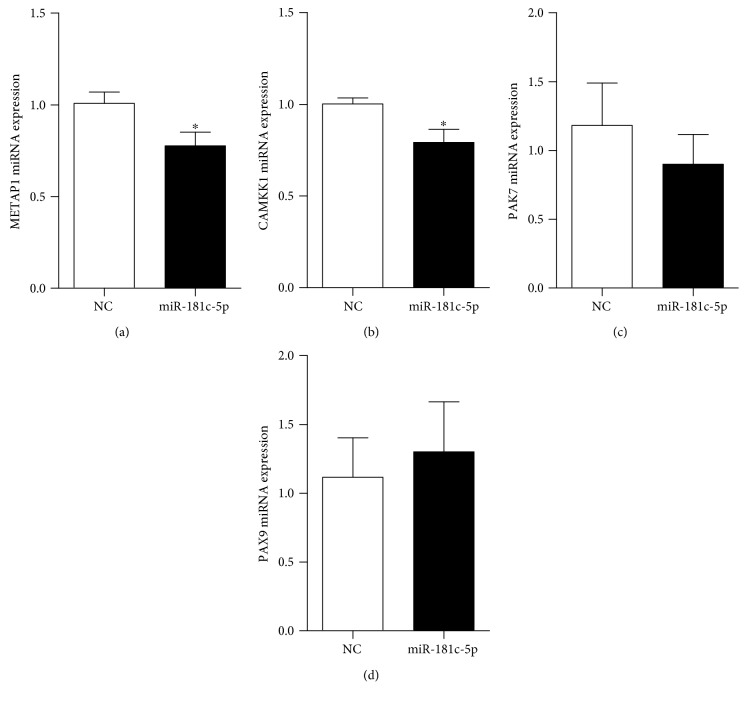
mRNA expression of METAP1 (a), CAMKK1 (b), PAK7 (c), and PAX9 (d) in H9C2 cardiomyocytes transfected with miRNA-181c-5p agomir (miR-181c-5p) or its negative control (NC). Data are shown as means ± SEM; ^∗^*P* < 0.05 vs. NC, *n* = 6.

**Figure 5:**
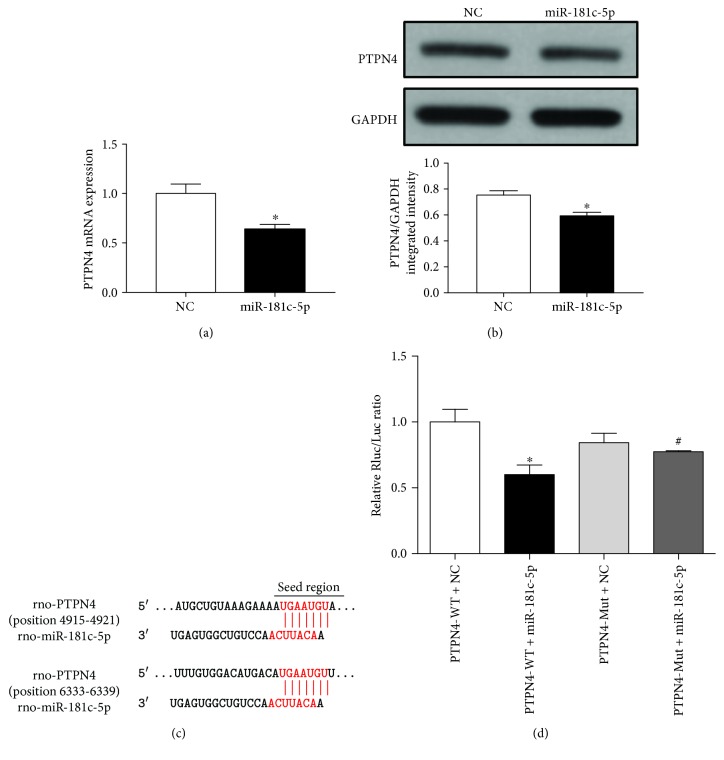
Overexpression of miR-181c-5p results in reduced levels of mRNA (a) and protein (b) of PTPN4 in H9C2 cardiomyocytes. (c) Potential target sites for miR-181c-5p binding in the 3′-UTR of PTPN4 mRNA (rat), as predicted by the TargetScan program. (d) Luciferase assays identified PTPN4 as a direct target of miR-181c-5p. Data are shown as means ± SEM; ^∗^*P* < 0.05 vs. NC or PTPN4-WT+NC, ^#^*P* < 0.05 vs. PTPN4-WT+miR-181c-5p, *n* = 5.

The abovementioned results indicated the importance of miR-181c-5p in H/R-induced cell injury and apoptosis in H9C2 cardiomyocytes. However, the underlying mechanism remained unknown. To predict the potential binding sites of miR-181c-5p, different computer algorithms (TargetScan, miRDB) were used to scan the 3′-UTR of mRNAs for potential genes. These algorithms generate hundreds of putative targets. To limit the scope, there were 46 genes chosen by the overlap of predicted targets of miR-181c-5p *via* TargetScan and miRDB analysis (Table 2). Next, we searched the functions of these 46 genes through GeneCards database and checked if any of the genes are functionally related to the cell death, cell cycle, or cell survival. Finally, these five potential targets, including protein tyrosine phosphatase nonreceptor type 4 (PTPN4) [24], methionyl aminopeptidase 1 (METAP1) [25], calcium/calmodulin-dependent protein kinase kinase 1 (CAMKK1) [26], p21 protein (Cdc42/Rac)-activated kinase 7 (PAK7) [27], and paired box 9 (PAX9) [28], were further confirmed by the publications in PubMed database, which are closely associated with cell apoptosis pathway. The miRNA functions to negatively regulate the expression of the target mRNA at the posttranscriptional level by promoting the mRNA cleavage or at the translational level by serving as a translational repressor to inhibit the translation of protein from mRNA or both [29]. Thus, the mRNA level of these five potential targets was detected by real-time PCR in the H9C2 cardiomyocytes transfected with miR-181c-5p agomir. Among these genes, the mRNA levels of METAP1, CAMKK1, PAK7, and PAX9 displayed few or no reduction after miR-181c-5p agomir transfection (METAP1 (23%), CAMKK1 (21%), PAK7 (12%), and PAX9 (2%); Figure 4), while PTPN4 is the one which showed the lowest mRNA level (by 36% reduction) (Figure 5(a)). Consistently, after transfection with miR-181c-5p agomir, there was significantly reduced protein level of PTPN4 as compared to the cells transfected with NC-agomir (Figure 5(b)). However, the protein expression of METAP1 and CAMKK1 was not significantly changed in miR-181c-5p agomir-transfected H9C2 cardiomyocytes (data not shown). From the results of TargetScan program prediction, the complementary sequence of miRNA-181c-5p was located on the positions from 4915 to 4921 or from 6333 to 6339 (or both) on the 3′-UTR of rat PTPN4 mRNA and there were 7 pairs of Watson-Crick match (A-U pair or G-C pair) (Figure 5(c)). Furthermore, luciferase reporter assay confirmed that miR-181c-5p led to a significant reduction in luciferase activity for the wild-type 3′-UTR construct. However, such reduction of luciferase activity was cancelled when the miR-181c-5p binding site was mutated, indicating that PTPN4 is a direct target gene of miR-181-5p (Figure 5(d)).

### 3.5. Knockdown of PTPN4 Reproduced the Proapoptotic Effect of miR-181c-5p

**Figure 6:**
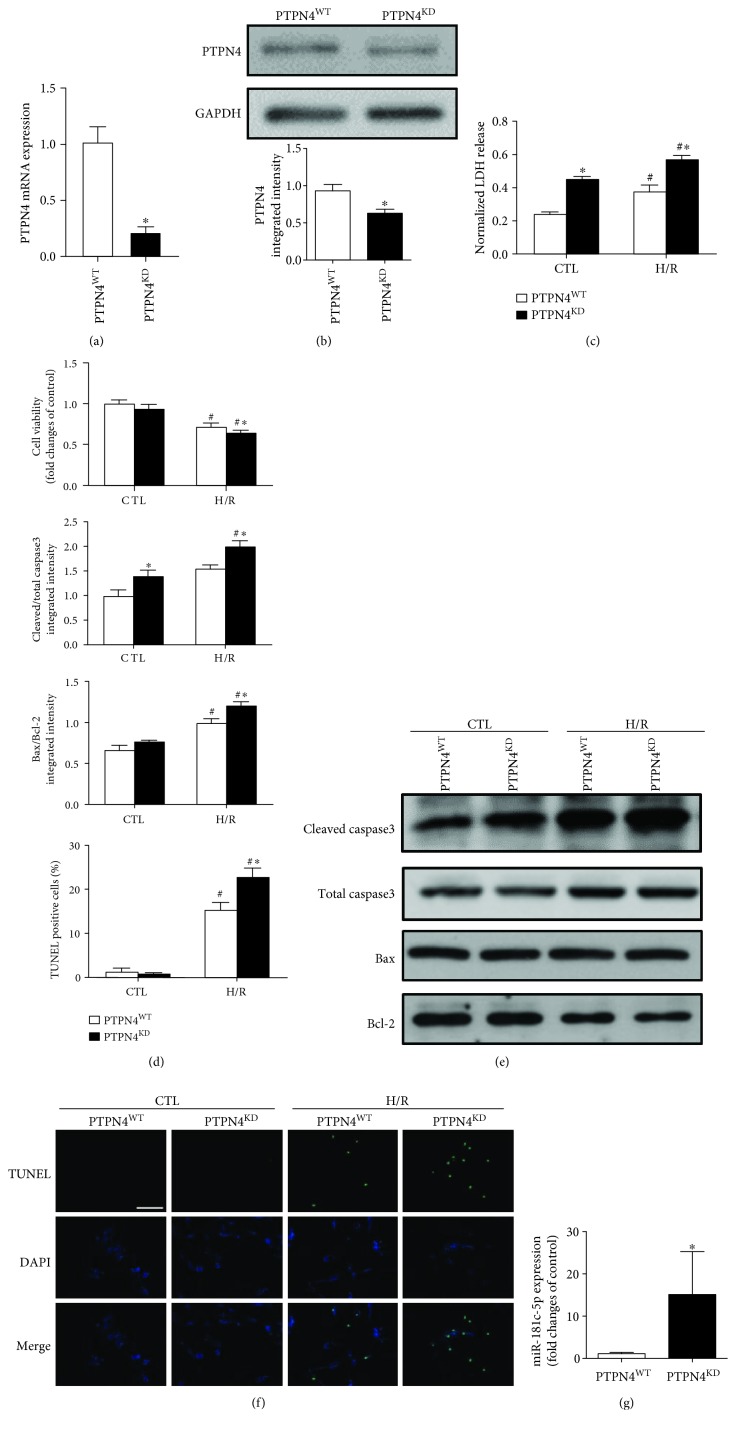
Transfection of cells with PTPN4 siRNA (PTPN4^KD^) resulted in significant reduction of PTPN4 mRNA (a) and protein (b) levels in H9C2 cardiomyocytes. Knockdown of PTPN4 exacerbated the H/R-induced H9C2 cell injury, as evidenced by further increased LDH release (c), reduced cell viability (d), and increased apoptotic cell death as evidenced by increased cleaved caspase 3 (e) and increased TUNEL-positive cells (f). Scale bar: 200 *μ*m. Knockdown of PTPN4 significantly increased the expression of miR-181c-5p (g). Data are shown as means ± SEM; ^#^*P* < 0.05 vs. CTL, ^∗^*P* < 0.05 vs. scramble siRNA (PTPN4^WT^), *n* = 6.

Although emerging evidence has demonstrated that PTPN4 regulates neuronal cell homeostasis by protecting neurons against apoptosis [30], it is still unclear whether or not PTPN4 protects cell apoptosis in cardiomyocytes. To further confirm whether or not knockdown of PTPN4 could mimic the proapoptotic effect of miR-181c-5p, siRNA technology was applied to reduce endogenous PTPN4 level in H9C2 cardiomyocytes. When compared to mock-transfected cells, there was a significant reduction of PTPN4 mRNA level (Figure 6(a)) and protein level (Figure 6(b)) in H9C2 cardiomyocytes transfected with PTPN4 siRNA (siPTPN4). Knockdown of PTPN4 significantly increased LDH release before and after H/R stimulation (Figure 6(c)). The knockdown of PTPN4 did not influence the cell viability at the basal level but significantly reduced H/R-stimulated reduction of cell viability (Figure 6(d)), indicating that PTPN4 knockdown aggravates H/R-induced cell injury. Furthermore, the knockdown of PTPN4 significantly increased the H/R-induced enhancement of ratios of cleaved/total caspase 3 and Bax/Bcl-2 (Figure 6(e)) and the number of TUNEL-positive cells (Figure 6(f)). Collectively, these data suggested that miR-181c-5p may aggravate H/R-induced cell injury and apoptosis by targeting PTPN4 expression in H9C2 cardiomyocytes. In addition, the miR-181c-5p expression was significantly increased in the siPTPN4-transfected H9C2 cells (Figure 6(g)), indicating the existence of a negative feedback loop between miR-181c-5p and PTPN4 expression, the greater miR-181c-5p expression further aggravates H/R-induced cell injury and apoptosis.

## 4. Discussion

Cardiomyocyte apoptosis plays a significant role in the pathology of cardiac damage in the context of myocardial I/R injury, which results in the loss of cardiomyocyte volume and the subsequent cardiac diastolic/systolic dysfunction [31]. Thus, examining ways to attenuate I/R-induced cardiomyocyte apoptosis is of clinical interest to combat myocardial I/R injury. The present study demonstrated that the expression of miR-181c-5p but not miR-181c-3p was significantly upregulated in the myocardium and H9C2 cardiomyocytes in response to I/R injury and H/R stimulation, respectively. Our findings are in line with several previous studies, which showed that miR-181c-5p level was significantly upregulated in subjects with heart failure or under hypoxia condition [13, 16]. The upregulation of miR-181c-5p was associated with enhanced H/R-stimulated cell injury and apoptosis in H9C2 cardiomyocytes, suggesting that enhancement of miR-181c-5p may be involved in the pathology of myocardial I/R injury. Indeed, overexpression of miR-181c-5p aggravated H/R-induced cell injury (increased LDH level and reduced cell viability) and exacerbated H/R-induced cell apoptosis (increased Bax/Bcl-2 ratio, greater cleaved caspase 3 expression, and more TUNEL-positive cells). In contrast, inhibition of miR-181c-5p significantly suppressed H/R-stimulated increment of LDH release, cleaved caspase-3 expression, and apoptotic cell death. Taken in conjunction, these findings suggest that miR-181c-5p exerts proapoptotic effect in cardiomyocytes in response to H/R stimulation.

Previous studies indicated that miR-181c-5p directly targeted the 3′-untranslated region of Bcl-2, one of the antiapoptotic proteins, aggravated tumor necrosis factor- (TNF-) *α*-induced cell apoptosis, and regulated mitochondrial morphology in neonatal rat cardiomyocytes [32]. Results of the present study conducted on H9C2 cardiomyocytes are in line with the observation that miR-181c-5p exerts proapoptotic effect. However, Bcl-2 could not represent the potent target of miR-181c-5p in H9C2 cardiomyocytes, as overexpression of miR-181c-5p did not result in the reduction of Bcl-2 expression in nonstimulated H9C2 cardiomyocytes. There are several possible explanations regarding the discrepancy between our and others' study: firstly, in Wang's study, the stimulus TNF*α* (10 ng/mL) was used to treat ventricular myocytes for one hour to induce cell apoptosis through the extrinsic signaling pathway by binding to the TNF receptor on the cell membrane [33]. However, in the present study, instead of using primary cardiomyocytes, H9C2 cardiomyocytes were treated with H/R (6 hours/6 hours) to induce cell apoptosis, which include both the intrinsic and extrinsic pathways [4]. Thus, different cell types and different stimuli may lead to discrepancy between our and others' study. In addition, instead of using miR-181c-5p mimics, chemically modified double-strand miRNA mimics, miR-181c-5p agomir was used to establish the overexpression model in the present study (~6000-fold vs. ~8-fold in Wang's study), which may also result in discrepancy between our and others' study. Taken together, Bcl-2 may not be the downstream signaling molecule that mediates the proapoptotic effect of miR-181c-5p in the current experimental setting.

The next challenge for us is to identify other potential mRNA target(s) that can account for the proapoptotic effect of miR-181c-5p in H/R-stimulated H9C2 cardiomyocytes. By using computational prediction algorithms, PTPN4 was predicted as a target gene of miR-181c-5p. PTPN4 is a nonreceptor tyrosine phosphatase containing an N-terminal FERM (band 4.1, ezrin, radixin, and moesin) domain, a PDZ (PSD-95/Dlg/ZO-1) domain, and a C-terminal catalytic tyrosine phosphatase (PTP) domain, which is localized in the cytoplasm and at the plasma membrane [24]. After activation by trypsin and calpain, PTPN4 is cleaved and the active form of PTPN4 consists of PDZ and PTP domains [24, 34]. Studies have demonstrated that PTPN4 regulates neuronal cell homeostasis by protecting neurons against apoptosis [30]. In neuroblastoma and glioblastoma cell lines, mitogen-activated protein kinase p38*γ* served as a cellular partner of PTPN4 [35]. This interaction is mediated by the binding of C terminus of p38*γ* to the PDZ domain of PTPN4, which antagonizes the catalytic autoinhibition of PTPN4, leading to cell apoptosis [24, 35]. Indeed, silencing of PTPN4 or p38*γ* gene leads to massive cell death in human osteosarcoma cells [36]. However, to our knowledge, there is no study that has investigated whether or not PTPN4 protects cell apoptosis in cardiomyocytes in the past. In the present study, knockdown of PTPN4 mRNA reproduced the proapoptotic effects of overexpression of miR-181c-5p in H9C2 cardiomyocytes, such as enhanced cleaved caspase 3 expression, greater ratio of Bax/Bcl-2, and increased positive TUNEL apoptotic cells, suggesting that PTPN4 could protect against H/R-induced cell apoptosis in H9C2 cardiomyocytes. Furthermore, multiple lines of evidence supports PTPN4 to be the target of miR-181c-5p in cardiomyocytes as demonstrated by the findings that (1) the seed sequence of miR-181c-5p displays perfectly complementary matching with the 3′-UTR of the rat PTPN4 gene; (2) overexpression of miR-181c-5p significantly suppressed PTPN4 expression at the transcriptional (mRNA) and posttranscriptional (protein) level; and (3) reduced luciferase activity for the cotransfection with wild-type PTPN4 plasmid and miR-181c-5p agomir but had no effect when the binding sites were mutated. Taken in conjunction, these findings collectively indicated that miR-181c-5p may target PTPN4 and therefore aggravate H/R-induced activation of apoptosis in H9C2 cardiomyocytes. Interestingly, our study demonstrated that the miR-181c-5p expression was significantly increased in the siPTPN4-transfected H9C2 cardiomyocytes, suggesting the existence of a negative feedback loop between miR-181c-5p and PTPN4 expression. It is thus apparent that in response to H/R stimulation, the enhanced miR-181c-5p downregulates the protein expression of PTPN4, and the reduction of the latter in turn triggers greater expression of miR-181c-5p in H9C2 cardiomyocytes and aggravates H/R-induced cell injury and apoptosis.

In addition to the role of PTPN4 in protecting cell apoptosis, PTPN4 can suppress the tyrosine phosphorylation and cytoplasm translocation of TRAM, which in turn lead to the imbalance of TRAM-TRIP interaction. Thus, PTPN4 may specifically inhibit TRIF-dependent TLR4 and its downstream NF*κ*B signaling cascade [37]. Numerous studies have demonstrated that TLR4/NF*κ*B-mediated proinflammatory responses exacerbate cardiomyocyte death in the context of myocardial I/R injury [38]. The expression of TLR4 and NF*κ*B was significantly increased in the experimental model of myocardial I/R injury when compared to the sham operation group [39]. Thus, it is possible to hypothesize that miR-181c-5p may target PTPN4 and aggravate H/R-induced cell injury via activation of TLR4/NF*κ*B signaling pathway. However, this hypothesis is beyond the scope of the present study and warrants investigation in future studies.

It should be noted that besides miR-181c-5p many other miRNAs were also affected during the course of myocardial I/R injury. For example, (1) H/R (2-hour hypoxia followed by 24-hour reoxygenation) significantly resulted in the reduction of miR-125b level in H9C2 cells, and overexpression of miR-125b protected the myocardium against I/R injury by preventing p53-mediated apoptotic cell death and suppressing TRAF6-mediated NF*κ*B activation [40]. (2) After myocardial ischemia for 30 minutes followed by 2 hours of reperfusion, the expression of miR-93 was remarkably downregulated in the rat myocardium. Transfection of miR-93 mimics into the heart protected against I/R-induced cardiomyocyte apoptosis by inhibiting PI3K/AKT/PTEN signaling [41]. (3) The level of miR-1 in the heart was significantly upregulated during myocardial I/R injury; overexpression of miR-1 aggravated cardiac I/R injury *via* inhibiting prosurvival proteins, such as PKC*ε* and HSP60 [42]. Thus, in response to I/R injury or H/R stimulation, the expression of several miRNAs would be differentially modulated in a spatial and temporal manner to protect or exacerbate myocardial I/R injury at multiple levels. Although it is still unclear whether or not some miRNAs directly alter the expression of other miRNAs in pathophysiological states, the current study confirms the importance of miR-181c-5p in its unique ability to exacerbate H/R-induced cell apoptosis *via* targeting PTPN4 in H9C2 cardiomyocytes.

## 5. Conclusion

In summary, the present study demonstrates that I/R injury and H/R stimulation can induce the expression of miR-181c-5p in the myocardium and H9C2 cardiomyocytes, respectively. Whether or not miR-181c-5p can be applied as a biomarker for ischemic heart disease warrants further investigation. In addition to serve as a diagnostic tool, our current work demonstrates that through targeting PTPN4 miR-181c-5p exacerbates H/R-induced cell apoptosis and cell injury in H9C2 cardiomyocytes. miR-181c-5p may have a potential clinical use to develop viable targets for the pharmacological intervention of ischemic heart disease.

## Figures and Tables

**Table 1:** Primers used in quantitative real-time polymerase chain reactions.

Gene	Forward sequence 5′-3′	Reverse sequence 5′-3′
GAPDH	GGGTGTGAACCACGAGAAAT	ACTGTGGTCATGAGCCCTTC
PTPN4	CCCTCTTCCCCTGAAAAGTC	TCATGGGTGTGTTCTGCAAT
METAP1	GCCCGTTTTGTTTTGAGTGT	GACGGGCAGATTTAGGTCAA
CAMKK1	GGTCAGCGAGGAACTCAAAG	CCAAAGGAACGCTTTCTCAG
PAK7	CCACCGCTTCTTACTTGAGC	CCAAATATTCCCTGGGGTCT
PAX9	GCTGTTGCATTAGCCTCCTC	AAAACAGAAAGCCAGGAGCA

GAPDH = glyceraldehyde-3-phosphate dehydrogenase; PTPN4 = protein tyrosine phosphatase nonreceptor type 4; METAP1 = methionyl aminopeptidase 1; CAMKK1 = calcium/calmodulin-dependent protein kinase kinase 1; PAK7 = p21 protein (Cdc42/Rac)-activated kinase 7; PAX9 = paired box 9.

**Table 2:** Predicted targets of miR-181c-5p *via* overlap of TargetScan and miRDB analysis.

Ortholog of target gene	Gene name
CTTNBP2NL	CTTNBP2 N-terminal like
RLF	Rearranged L-myc fusion sequence
ZFAND5	Zinc finger, AN1-type domain 5
CPD	Carboxypeptidase D
ATP1B1	ATPase, Na+/K+ transporting, beta 1 polypeptide
LRP12	Low-density lipoprotein-related protein 12
MAP1B	Microtubule-associated protein 1B
PAWR	PRKC, apoptosis, WT1, regulator
AKIRIN1	Akirin 1
HMGB2	High-mobility group box 2
RAN	RAN, member RAS oncogene family
RAD21L	RAD21-like (S. pombe)
SNN	Stannin
TRAK1	Trafficking protein, kinesin-binding 1
UBP1	Upstream-binding protein 1
E2F7	E2F transcription factor 7
SEL1L	Sel-1 suppressor of lin-12-like (C. elegans)
TBL1XR1	Transducin (beta)-like 1X-linked receptor 1
ZFHX4	Zinc finger homeodomain 4
ZFAND6	Zinc finger, AN1-type domain 6
DERL1	Der1-like domain family, member 1
*PAK7*	p21 protein (Cdc42/Rac)-activated kinase 7
ELAVL2	ELAV (embryonic lethal, abnormal vision, Drosophila)-like 2
*PAX9*	Paired box 9
GPSM1	G-protein signaling modulator 1 (AGS3-like, C. elegans)
ZDHHC7	Zinc finger, DHHC domain containing 7
CARM1	Coactivator-associated arginine methyltransferase 1
SMAD7	SMAD family member 7
*PTPN4*	Protein tyrosine phosphatase nonreceptor type 4
TGIF2	TGFB-induced factor homeobox 2
SEC24C	Sec24 related gene family, member C (S. cerevisiae)
COL16A1	Collagen, type XVI, alpha 1
GOLGA1	Golgi autoantigen, golgin subfamily a, 1
*METAP1*	Methionyl aminopeptidase 1
RFX5	Regulatory factor X, 5 (influences HLA class II expression)
EVI2A	Ecotropic viral integration site 2a
KCNK10	Potassium channel, subfamily K, member 10
MEGF9	Multiple EGF-like domains 9
OGT	O-linked N-acetylglucosamine (GlcNAc) transferase
GREM1	Gremlin 1
MPZL3	Myelin protein zero-like 3
CDH23	Cadherin 23 (otocadherin)
ABHD3	Abhydrolase domain containing 3
*CAMKK1*	Calcium/calmodulin-dependent protein kinase kinase 1
WWC2	WW, C2 and coiled-coil domain containing 2
SPP1	Secreted phosphoprotein 1

## Data Availability

The data used to support the findings of this study are available from the corresponding authors upon request.

## Abbreviations

I/RI: Ischemia/reperfusion injury
H/R: Hypoxia/reoxygenation
H9C2: Rat origin cardiomyocytes
DMEM: Dulbecco's modified Eagle's medium
FBS: Fetal bovine serum
PS: Penicillin/streptomycin
CTL: Control
NC: Negative control
IS: Myocardial infarct size
TTC: 1% 2,3,5-triphenyltetrazolium chloride
AAR: The area at risk
siRNA: Small interfering RNA
PCR: Polymerase chain reaction
LDH: Lactate dehydrogenase
MTT: 3-(4,5-Dimethylthiazol-2-yl)-2,5-diphenyltetrazolium bromide
GAPDH: Glyceraldehyde-3-phosphate dehydrogenase
Bcl-2: B cell lymphoma/lewkmia-2
Bax: Bcl-2-associated X protein
TUNEL: Terminal deoxynucleotidyl transferase dUTP nick end labeling.
